# Determining *Clostridium difficile* intra-taxa diversity by mining multilocus sequence typing databases

**DOI:** 10.1186/s12866-017-0969-7

**Published:** 2017-03-14

**Authors:** Marina Muñoz, Dora Inés Ríos-Chaparro, Manuel Alfonso Patarroyo, Juan David Ramírez

**Affiliations:** 1 Grupo de Investigaciones Microbiológicas–UR (GIMUR), Programa de Biología, Facultad de Ciencias Naturales y Matemáticas, Universidad del Rosario, Carrera 24 # 63C - 69, Bogotá, Colombia; 2 Posgrado Interfacultades Doctorado en Biotecnología, Facultad de Ciencias, Universidad Nacional de Colombia, Bogotá, Colombia; 3 Molecular Biology and Immunology Department, Fundación Instituto de Inmunología de Colombia (FIDIC), Bogotá, Colombia; 4 School of Medicine and Health Sciences, Universidad del Rosario, Bogotá, Colombia

**Keywords:** *Clostridium difficile*, Multilocus sequence typing (MLST), Distribution pattern, Population structure

## Abstract

**Background:**

Multilocus sequence typing (MLST) is a highly discriminatory typing strategy; it is reproducible and scalable. There is a MLST scheme for *Clostridium difficile* (CD), a gram positive bacillus causing different pathologies of the gastrointestinal tract. This work was aimed at describing the frequency of sequence types (STs) and Clades (C) reported and evalute the intra-taxa diversity in the CD MLST database (CD-MLST-db) using an MLSA approach.

**Results:**

Analysis of 1778 available isolates showed that clade 1 (C1) was the most frequent worldwide (57.7%), followed by C2 (29.1%). Regarding sequence types (STs), it was found that ST-1, belonging to C2, was the most frequent. The isolates analysed came from 17 countries, mostly from the United Kingdom (UK) (1541 STs, 87.0%). The diversity of the seven housekeeping genes in the MLST scheme was evaluated, and alleles from the profiles (STs), for identifying CD population structure. It was found that *adk* and *atpA* are conserved genes allowing a limited amount of clusters to be discriminated; however, different genes such as *drx*, *glyA* and particularly *sodA* showed high diversity indexes and grouped CD populations in many clusters, suggesting that these genes’ contribution to CD typing should be revised. It was identified that CD STs reported to date have a mostly clonal population structure with foreseen events of recombination; however, one group of STs was not assigned to a clade being highly different containing at least nine well-supported clusters, suggesting a greater amount of clades for CD.

**Conclusions:**

This study shows the usefulness of CD-MLST-db as a tool for studying CD distribution and population structure, identifying the need for reviewing the usefulness of *sodA* as housekeeping gene within the MLST scheme and suggesting the existence of a greater amount of CD clades. The study also shows the plausible exchange of genetic material between STs, contributing towards intra-taxa genetic diversity.

**Electronic supplementary material:**

The online version of this article (doi:10.1186/s12866-017-0969-7) contains supplementary material, which is available to authorized users.

## Background

Multilocus sequence typing (MLST) is a highly discriminatory method for the molecular identification of microorganism genotypes based on the nucleotide sequence of internal fragments of multiple, highly conserved, single copy housekeeping genes [1]. Housekeeping genes’ polymorphisms allow the assignation of alleles which, as a combination of at least seven targets, define the allele profile or sequence type (ST), which represents each genotype [2]. There is a standardised scheme for different bacterial and fungal species whose sequences are deposited in free-access databases, thereby enabling the results obtained from different regions of the world to be compared. These characteristics consolidate the MLST strategy as a reproducible and scalable typing system, contributing uniform nomenclature for intra-taxa classification [2]. In addition to its usefulness as a typing tool, the analysis of data generated from the MLST scheme (‘MLSA’ for multilocus sequence analysis), provides information about evolutionary and population biology, mainly between clonal populations, due to its great power for group discrimination [3]. Regarding bacteria, the MLSA strategy describes genetic relationships between genotypes, geographic and temporal infection patterns for pathogenic genotypes (associated with outbreaks of disease) [4, 5].

There is a MLST scheme for *Clostridium difficile* (CD), an anaerobic, spore-forming, gram-positive bacillus which colonises the gastrointestinal tract through oral-faecal contact [6, 7]. CD infection is related to a broad range of manifestations, including asymptomatic colonisation, clinical pictures of slight to serious diarrhoea in individuals having disequilibrium in their gastrointestinal microbiota (as happens in populations exposed to antibiotics), to serious diseases such as pseudomembranous colitis, toxic megacolon, perforations of the colon and even a patient’s death [8]. The impact of CD infection has increased during the last 15 years, due mainly to the emergence of hypervirulent strains [8], causing serious outbreaks around the world [9]. These strains are characterised by: leading to clinical pictures having greater severity (due to toxin hyper-production) [10], failure regarding therapeutic management (due to the presence of point mutations or cassettes, associated with resistance to antibiotics) [11] and high rates of recurrence (related to increased sporulation) [12].

The emergence of these hypervirulent strains has been seen to favour the CD genome’s dynamic architecture, having a high percentage of mobile genetic elements (around 11% of the CD-630 reference strain’s genome [13]). The presence of these elements has led to an increase in their genetic diversity and mobilisation of accessory genes (due to horizontal transfer), which could contribute to CD’s success as an opportunistic pathogen [14]. The association between the molecular characteristics and the clinical pictures produced by CD infection has led to different strategies aimed at describing epidemiological profiles, genetic diversity and CD evolution using three approaches. One involves the microanalysis of the genome’s architecture, such as restriction endonuclease analysis (REA) or pulsed-field gel electrophoresis (PFGE); another involves identifying specific regions in the genome, such as PCR ribotyping, and toxin-typing whilst a third involves sequencing strategies, the MLST scheme being extremely useful. The aforementioned approaches can be combined for taxonomic classification of CD [14], i.e. the hypervirulent strain BI/NAP1/027, for REA and PFGE groups and PCR ribotyping [15].

The consensus MLST scheme for CD was developed by David Griffiths, Kate Dingle (University of Oxford, UK) et al., in 2010 [16]. This scheme is based on the following housekeeping genes: *adenylate kinase (adk)*, *ATP synthase subunit alpha (atpA)*, *1-deoxy-D-xylulose 5-phosphate reductoisomerase (dxr)*, *serine hydroxymethyltransferase (glyA)*, *recombinase A (recA)*, *superoxide dismutase (sodA)* and *triose phosphate isomerase (tpi).* The sequences of these seven housekeeping genes from isolates around the world are deposited in the centralised database (http://pubmlst.org/cdifficile/ [17]) and have allowed to identify more than 300 genotypes,

Phylogenetic analysis based on the concatenated sequences of the seven markers for the STs assignment, from the representative set of isolates (50 reference + 102 clinical isolates, from different sources) used for the development of MLST scheme [16], allowed to identify four main phylogenetic lineages, called clades (1 to 4), and one outlier (Conformed exclusively by the ST-11; PCR ribotype 078). In parallel to the advances in the description of intra-taxa diversity, the results of the evolutionary studies have shown the correlation between genome’s dynamic and different outcomes (including toxin-production profiles and clinical impact) [18].

The most representative molecular epidemiology study of CD, developed by Dingle et al., in 2011 [19], included 1,290 isolates from a single geographic location (United Kingdom). This study confirmed the major clonality of CD populations (with occasional deviations possibly due to recombination effects), the existence of five main clades (C1 to C5, the latter consisting exclusively of ST-11) and the association of specific toxigenic variants with clades.

A recent review summarises the diversity and evolutionary features of CD genome [14]. This report describes atypical arrangements between strains belonging to C5 clade and suggests the existence of additional clades such as C-I clade. Each clade has particular features related to ribotyping (RT) profiles and toxin production, finding that **C1** represents a highly heterogeneous cluster (including the largest number of isolates, >100 STs), having toxigenic and non-toxigenic members. The STs belonging to this group are the most frequently detected in CD-infected patients, ST2, 14 and 49 (RT014), ST9 (RT002) and ST17 (RT018) having the greatest clinical impact. C1 STs are characterised by being positive for the two main toxins (A and B) (TcdA+/TcdB+) but negative for binary toxin (CDT). **C2** includes the hypervirulent ribotype RT027 (ST1 and others related to it, such as ST41 (RT244), positive for the three toxins (TcdA+/TcdB+/CDT+)). **C3**, even though not having been studied in detail, has STs isolated from humans (mainly in Europe) having positive toxigenic profiles for three toxins (TcdA+/TcdB+/CDT+) (toxigenic strains). **C4** includes strains exclusively for TcdB, but having a great impact at clinical level, associated with the appearance of outbreaks in various regions around the world, i.e. ST37 (RT017). C4 strains have shown resistance to clindamycin and fluoroquinolones. **C5** is the most heterogeneous, being related to infections in different sources (humans, animals and food) worldwide and displays atypical toxinogenic profiles [20]. Interestingly, the sixth clade “C-I” is highly divergent and apparently non-toxigenic. This report provides clues about the existence of a greater number of genetic groups than the ones described to date.

Taking into account the usefulness of MLST for genotyping, multilocus sequence analysis (MLSA) should be carried out as a high resolution tool for evaluating phylogenetic relationships of prokaryotic populations, from the sequences of loci used for MLST [3]. This study was thus aimed at describing the frequency of STs and clades, also to evaluate the intra-taxa diversity of CD using a collection of isolates that represents the total known diversity of CD currently reported in the CD MLST databases (CD-MLST-db).

## Methods

### Obtaining data

The dataset having information regarding the descriptive variables related to the epidemiological profiles of all CD isolates was exported from the isolate database available in CD-MLST-db (http://pubmlst.org/cgi-bin/bigsdb/bigsdb.pl?db=pubmlst_cdifficile_isolates). The allelic profile sequences were exported from the script available in the allele/sequence definition database which allows downloading in extended multi-FASTA format, compatible with software for phylogenetic and diversity analysis (http://pubmlst.org/cgi-bin/bigsdb/bigsdb.pl?db=pubmlst_cdifficile_seqdef). The databases were compared for verifying descriptive information regarding all STs reported to date for having information representing known CD diversity (main objective of CD-MLST-db). Concerning STs not available in exportable files, an individual search of the profiles was made via the locus/sequence definitions tool, extracting profile information reported by authors.

### Isolate distribution patterns

The breakdown fields' section of the isolate database dataset was explored for describing provenance and epidemiological information. Descriptive statistics were used for describing ST and CD clade distribution profiles according to country and year of report in CD-MLST-db (in terms of frequencies and percentages). STATA software package version 11 (Stata Corporation, College Station, TX) was used for statistical procedures.

### MLST scheme allele polymorphisms

MLST scheme housekeeping gene allele sequences (defined as each sequence/variant) were analysed (that for CD was proposed by Griffiths et al.) [16]; these sequences were downloaded via XMFA (Extended Multi-FASTA)/concatenated FASTA formats option avalaible in CD-MLST-db. An initial comparison was made via multiple sequence comparison by log-expectation (MUSCLE) [21]. Multiple sequence alignments were used for preliminary analysis directed at identifying the amount of polymorphisms in datasets by determining percentage identity (and thus difference) regarding total allele length for each marker.

Molecular Evolutionary Genetics Analysis software, version 7 (MEGA7), was used for determining the nucleotide composition of the alleles reported for each marker [22]. Nucleotide composition analysis highlighted variable sites (defined as positions containing at least two types of nucleotides: a site which is not variable is referred to as a constant site) and parsimony-informative sites (variable sites containing at least two types of nucleotides, at least two of them occurring with a minimum frequency of two). Microsoft Excel was used for calculating parsimony-informative sites per amount of variable sites rate to have comparable data between markers.

### Sequence diversity analysis

The allele sequences of all STs reported in CD-MLST-db was the starting point for diversity analysis, considered as diferent combinations which might be found for alleles per gene (allele profiles). Sequences were downloaded from CD locus/sequence definition script for this analysis; they were identified by the amount of STs and the clade to which they belonged, labelled by country and year of report.

DnaSP v5 software was used for analysing genetic diversity, evaluated from concatenated sequences related to housekeeping gene, country or clade [23] for identifying genetic polymorphism level and distribution by calculating haplotype (gene) diversity, nucleotide diversity (π) as the average number of nucleotide differences per site between two sequences and θ indexes (per site, as indicator of mutation rate per nucleotide site per generation), calculated from Eta (h) (the total number of mutations and S the number of segregating/polymorphic sites). Each index was reported with corresponding standard deviations. The π indexes for each set of data were compared for identifying the category having the greatest diversity.

### Phylogenetic analysis

The concatenated sequences regarding the total amount of STs (n: 380) were initially compared via phylogenetic reconstruction based on Neighbour-Joining (NJ) algorithm. Subsequently, independent housekeeping genes were compared via multiple alignments using the MUSCLE algorithm. MEGA7 software was used for molecular phylogenetic analysis, using the maximum likelihood (ML) method based on the Tamura-Nei model [24]. The robustness of the nodes was evaluated by Bootstrap method using 1,000 replicates. Homologous genes in *Clostridium perfringens* were used for each housekeeping gene as outgroup sequences.

The number of clusters (bootstrap values equal or over 80.0%) for each housekeeping gene were identified and a colour was assigned. Cluster colours were then used to describe each ST. This analysis was the basis for constructing an allelic plot graphically representing the amount of clusters identified per housekeeping gene and the clusters to which each ST belonged in each of them. The results were contrasted with classification into clades traditionally accepted for CD, based on the seven housekeeping genes’ concatenated sequence discrimination power.

### Multilocus sequence analysis (MLSA)

Analyses of the population structure of CD were carried out using eBURST algorithm, which allows to identify groups of related isolates (clonal complexes/BURST groups) within MLST data set [24]. This analysis was developed using the plugin adapted for the BIGSdb database software, available in CD-MLST-db with the aim of determining shared alleles within clonal complexes per se. In order to identify rearrangements (loss and duplication events, hybridisation, horizontal gene transfer or recombination) between the sequences, phylogenetic networks were developed using Neighbor-Net method, available on SplitsTree4 (Version 4.14-4) package with bootstrap test of 1000 iterations [25]. Lastly, evidence of recombination events was assessed through Dnasp 4.0 analysis. This software uses the next rapid and statistically efficient tests to measure the following estimators: Sk^2, calculates the variance of the average number of nucleotide differences between pairs of sequences; Φ, product of effective population size and neutral mutation rate per site, C/μ, the ratio between per-site recombination rate and per-site mutation rate; and RM, quantifies the minimum number of recombination events in the history of the sample [26]. Neighbor-Net method and recombination analysis were carried out for both, the concatenated alignment and each housekeeping gene.

## Results

### General information regarding CD isolates

The information available in the isolate database covers 1597 isolates, representing the total diversity of CD reported globally (updated: 09-12-16). This database’s breakdown section has information about the source where isolates were obtained, most from humans (94.8% - n: 1,514) and (although in lesser numbers) animals, specifically canines and swine (2.9% - n: 47), clinical areas (2.1% - n: 34) and environmental (0.1% - n: 2). No information was found regarding the results of traditional typing strategies such as PFGE or RAE in most of the isolates reported. Concerning a description of genes encoding toxins (toxin typing), only binary toxin information was found regarding 1,432 isolates, from which 39.1% (n: 560) being positive for these genes and 60.9% were negative (n: 872), the remaining 142 isolates with epidemiological information were reported as “unassigned” for this toxin. No information was found regarding the main toxins (*tcdA* and *tcdB*).

Revising the profile/sequence definition databases gave 1,371 sequences associated with 380 allelic profiles (updated: 08-12-16). Each allelic profile in the latter database corresponded to at least one isolate deposited in the isolate database. Comparing the STs in both databases led to identifying a set of 181 STs whose sequences had been reported but for which no data was available in the isolate database. A manual search was thus made individually for this group of STs via the “search profiles” option, giving the place and year of report (the only information available). Including this set of 181 STs led to increasing the set of STs to be analysed to 1,778; however, 6 data were excluded as the ST had not been defined (giving 1,772 for descriptive CD geographical and temporal distribution pattern analysis).

### CD geographical and temporal distribution patterns

**Fig. 1:**
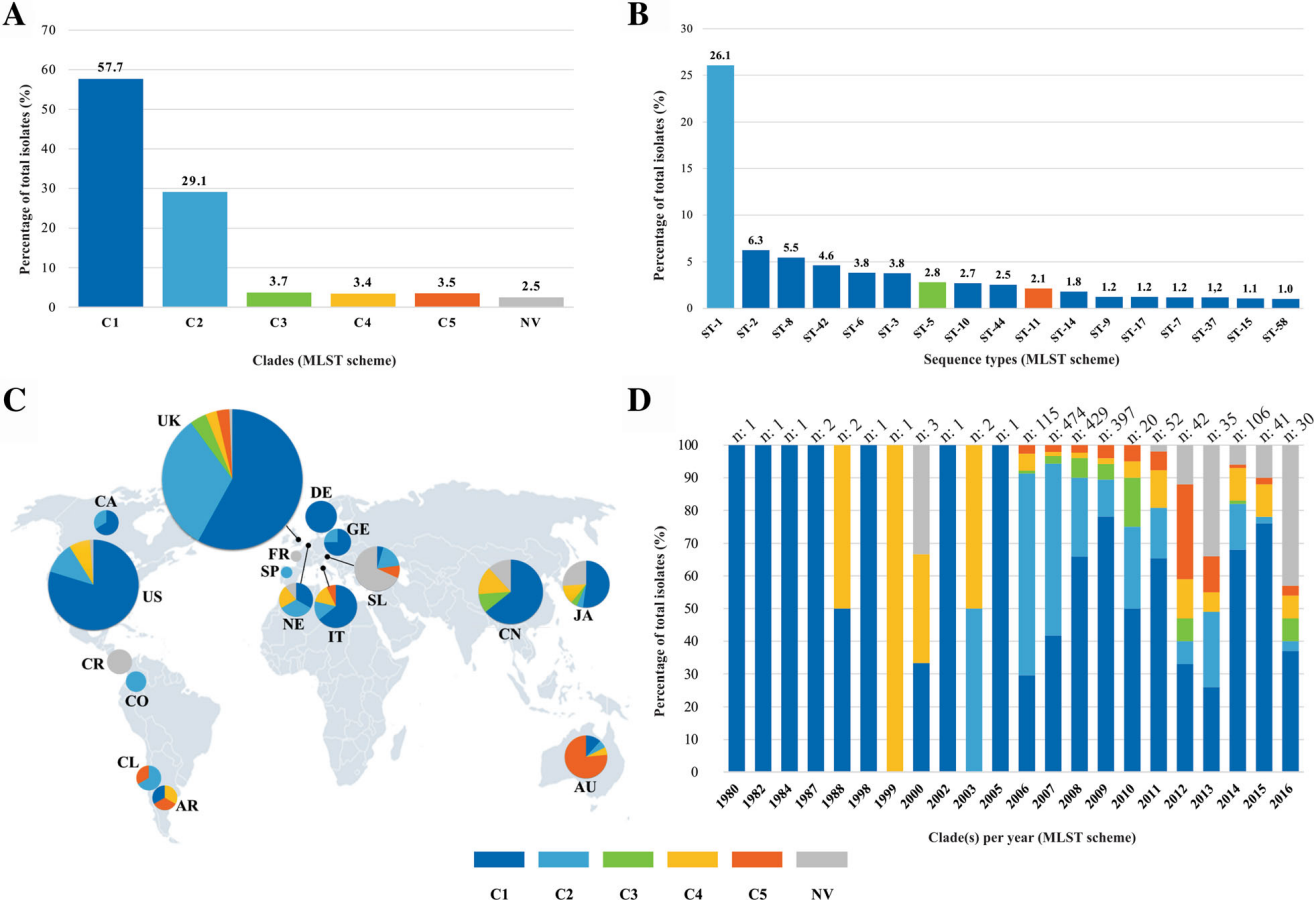
Epidemiological profiles of CD isolates reported in CD-MLST-db. **a** Frequency of clades to which all the STs reported belonged. **b** Frequency of main STs reported (only clades having ≥ 1.0% frequency are shown). **c** Geographical distribution of CD clades by country of origin. **d**. Temporal distribution (per year) by clade of reported STs. Percentages were calculated regarding all isolates included in the analysis (n: 1,772). The bottom map used was retrieved from https://www.google.com/about/datacenters/inside/locations/index.html

Complete set of isolates available in CD-MLST-db have been reported from 17 countries: Argentina (AR), Australia (AU), Canada (CA), Chile (CL), China (CN), Colombia (CO), Costa Rica (CR), Denmark (DE), France (FR), Germany (GE), Italy (IT), Japan (JA), Netherlands (NE), Slovenia (SL), Spain (SP), United Kingdom (UK) and United States (US). The greatest representation of isolates was from the UK (87.0% - n: 1,541), followed by, in order, the US (4.4% - n: 79), CN (2.4% - n: 42), JA (1.3% - n: 23), SL (1.2% - n: 22), AU (1.0% - n: 17) and IT (0.8% - n: 14); the other countries have reported less than 10 isolates each one. Regarding Latin-America, the database includes reports of isolates from AR (0.2% - n: 3), CL (0.2% - n: 3), CR (0.2% - n: 3) and CO (0.1% - n: 2). Most isolates (97.5% - n: 1,728) have been assigned to one of the five main clades (C1 to C5) used in the framework of the initial MLST scheme [16]; however, a group of 44 (2.2%) isolates was identified having no value assigned for a clade in the database (hereinafter no value: NV). CD clade distribution analysis identified C1 as being the most abundant (57.7% frequency; n: 1,023) in all samples, followed by C2 (29.1% frequency; n: 516). Most STs classified in the NV group came from SL (34.1% - n: 15) and UK (25.0% - n: 11) and to a lesser extent from JA (13.6% - n: 6), CN (11.4% - n: 5), US and NE (2.3% - n: 1, in both cases). Figure 1a describes the frequency of reported Clades in the set of analysed isolates.

Analyses of the STs reported in CD-MLST-db allowed us to identify that ST1, belonging to C2, was the most frequent (26.1% - n: 462), followed by a group of 16 STs (69.0% - n: 1,223), 13 STs belonging to C1. All the other STs have been reported as having lower than 1.0% frequency (≤1.0% of frequency – n ≤ 17). Figure 1b shows the frequencies for the main STs.

Analyses of distribution patterns of CD clades by country showed that C1 is the most frequent in most countries, except for CL, CO, CR, FR and SP, where this clade has not been reported. In the case of AU and SL others were the most frequent, being C5 and NV the most frequent and for CR where NV was predominant. Figure 1c shows the clade distribution profiles per country.

Clade distribution according to year of report in CD-MLST-db revealed an increase in reports from 2006 onwards (even though STs have been reported since 1980), most being reported from 2006 to 2009. Analysis of temporal variation per clade identified C1 as the most frequent, and had peaks during 2009/2010 and 2014/2015. Furthermore, in 2013 and 2016 an increase in the number of STs defined as NV was observed (Fig. 1d).

### MLST scheme allele diversity

An initial descriptive analysis of the sequences reported for each gene allowed to identify that the *glyA*, *tpi* and *sodA* genes showed the highest number of alleles (59, 58 and 54, respectively), compared to the other genes for which the number of alleles reported was below 46 (Additional file 1: Figure S1A). Multiple alignments of sequences for all alleles reported for each MLST scheme marker identified *adk* (89.8% identity), *atpA* (89.0%), *tpi* (86.1%) and *recA* (86.0%) genes with higher percentage of sequence identity. By contrast, the lower percentages of identity were found for *sodA* (77.3%), *dxr* (78.4%) and *glyA* (78.9%) (Additional file 1: Figure S1A). However, considering that not all the variable sites in the sequences were parsimony-informative sites, an informative index was calculated (according to that described in the methodology), i.e. comparable data amongst housekeeping genes. The results showed that the highest informative indexes were for *recA* (0.734) and *tpi* (0.725), compared to *adk* (0.686) having the lowest informative index (Additional file 1: Figure S1B). This analysis identified polymorphisms per position between the alleles reported for each marker. Additional file 2: Figure S2 shows the distribution of these polymorphisms throughout the length of these sequences, highlighting informative ones.

### Sequence diversity analysis

**Fig. 2:**
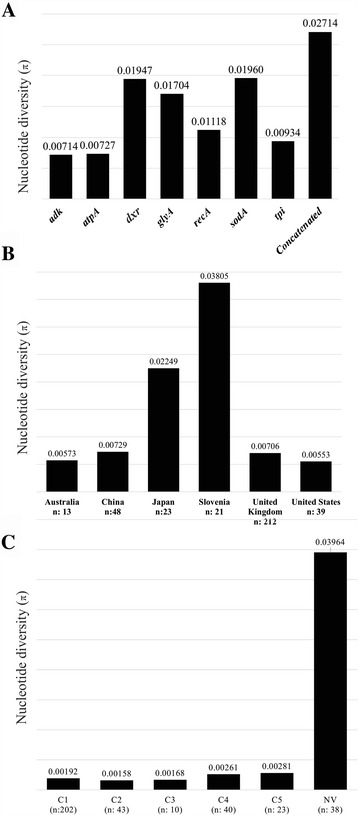
Graphic representation of nucleotide diversity (π) by: **a** housekeeping gene; **b** country and **c** clade

The genetic diversity analysis of the concatenated sequences of the seven housekeeping genes used for the MLST scheme was calculated for each gene and concatenated sequences. ST sequences were included in this analysis (380 reported in CD-MLST-db at the date of consultation). The results indicated that all indexes were greater for *sodA*, *dxr* and *glyA*, thereby agreeing with intra-taxon diversity findings. The results indicated that *sodA* and *dxr* were characterised by being the most diverse markers (Fig. 2a; Additional file 3: Table S1).

Diversity indexes were also calculated per country of provenance (including the total number of sequences reported for each country in each case); it was found that haplotype diversity was 1,000 for all cases, but isolates from SL (0.03805) and JA (0.02249) had considerably higher nucleotide diversity indexes regarding the other countries (0.007 indexes or less) (Fig. 2b; Additional file 4: Table S2).

Diversity indexes calculated per clade showed that clades traditionally reported for CD had <0.003 nucleotide diversity, ranging from 0.00158 (C2) to 0.00281 (C5). However, the STs included in the NV group had a considerably higher index (0.03964), thereby ratifying the greater diversity of STs forming this cluster (Fig. 2c). Additional file 5: Table S3 shows the set of diversity indexes per Clade. Figure 2a, b and c provide a graphical representation of nucleotide diversity indexes (π) regarding the different comparisons.

### Phylogenetic analyses and MLSA

Analysis of the population structure of CD (Based on BURST algorithm) allowed to identify a total of 14 BURST groups/Clonal Complexes (BG) and 24 STs singletons. BG 1 includes the highest number of STs and shows a central subgroup with four linked groups. The other groups are minority, with a maximum of two subgroups (BG-3, −5 and - 8) and only linked subgroups in the BG-8 (Additional file 6: Figure S3). The existence of linked subgroups could be a likely evidence of the existence of recombination events.

**Fig. 3:**
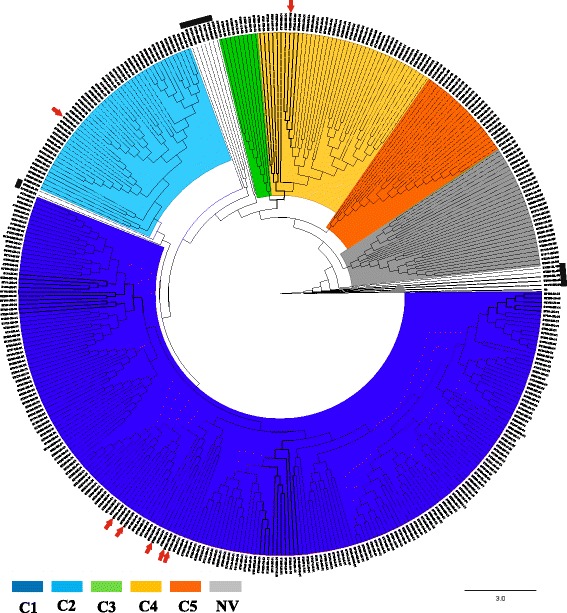
Phylogenetic analysis from concatenated sequences of the total set of STs (n: 380). The final tree was obtained with Neighbour Joining method using MEGA 7. The red arrows denote the STs with incongruence in their clustering. Black bars mark the STs that are not grouped in a single Clade/Cluster

NJ phylogenetic reconstruction was conducted based on the concatenated sequences, we observed the typical assignment of the six clusters (C1-C5 clades and NV cluster) and foreseen incongruences i.e. where C2, C3 and NV STs are grouped in C1 (Fig. 3) compatible with signals of recombination. Also, we observed some STs that are grouped within any clade/cluster.

To test the likelihood of recombination, we constructed Neighbour-Net networks, observing a tailored degree of reticulation for the case of C1-C4 and the independent clustering of C5 and NV in the concatenated network (Additional file 7: Figure S4). Furthermore, the independent analyses per each gene fragment was conducted where is observed that *sodA* and *recA* are highly divergent (Additional file 8: Figure S5).

**Fig. 4:**
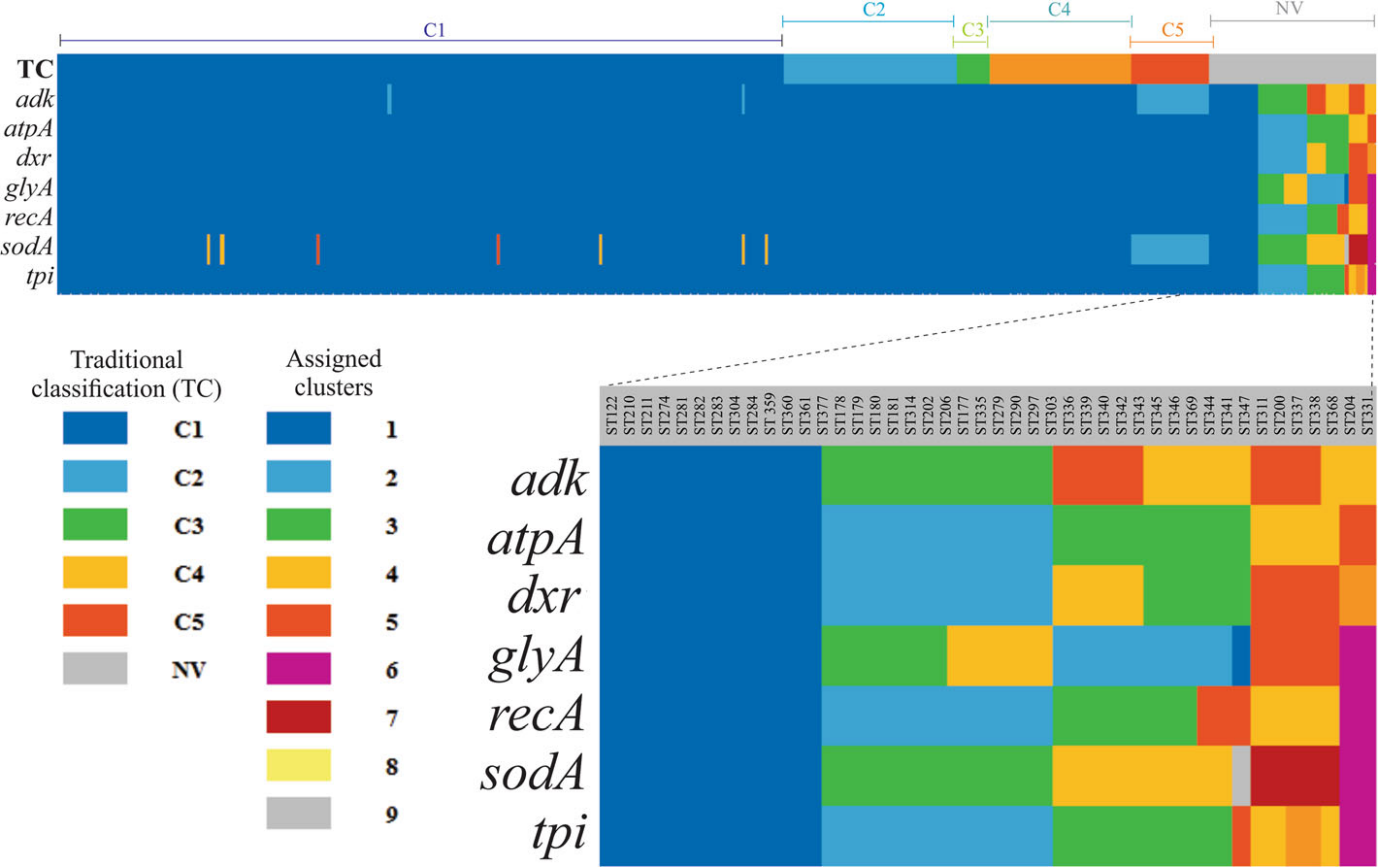
Allelic plot. Graphic representation of phylogenetic inferences where a colour is assigned to each cluster (Bootstrap ≥80.0%) found per gene (row). For each ST (columns) the cluster to which it belongs was analysed. The first row describes traditional classification per clade for CD. The upper section shows the results for all STs (n: 380) and the lower section shows the zoom of the STs defined as NV

Phylogenetic analysis from the sequences of STs for each housekeeping gene was used as the basis for determining taxon discrimination power. The number of clusters (bootstrap ≥80.0%) in each housekeeping gene were identified in the phylogenetic trees obtained and a colour was assigned for each of them. Additional file 9 shows the phylogenetic reconstructions for each gene from concatenated sequences obtained in MEGA 7 using ML. It was found that *adk* and *atpA* were housekeeping genes discriminating the least amount of taxa (5 each), contrary to that found for *sodA* and *dxr* (9 and 8 taxa, respectively). These findings agreed with diversity index results, confirming that *sodA* and *dxr* are highly polymorphic housekeeping genes. Figure 4 gives the classification results for each ST in the seven housekeeping genes, compared by allele plot construction. Three different analyses showed strong signals of recombination (Figs. 3, 4).

Therefore, we quantified these events through analyses by Dnasp 4.0, that showed a minimum of 119 recombination events in the evolutionary history of concatenated sequences. The per-site recombination rate was almost double (1.9147) in comparison to the per-site mutation (C/μ), accompanied by high parameters of both Sk ^ 2 (3781.796) and Φ (44.227), which confirms the high frequency of recombination events between the sequences used for the MLST scheme. When estimating recombination parameters for the independent genes it was found that *sodA* and *glyA* are the ones that contribute most to this behaviour (Table 1).

**Table 1:** Results of tests for the recombination based on Dnasp 4.0

	Sk^2	Φ	C/μ	RM
Concatenated	3781.796	44.227	1.9147	119
*adk*	31.714	3.575	2.2387	12
*atpA*	28.769	4.034	1.5664	12
*dxr*	126.315	8.011	1.8696	18
*glyA*	157.289	8.791	1.9388	25
*recA*	100.98	6.306	2.4035	14
*sodA*	178.515	8.802	2.2128	25
*tpi*	25.33	4.709	0.966	9

Sk^2: variance of the average number of nucleotide differences between pairs of sequences

Φ: product of effective population size and neutral mutation rate per site

C/μ: ratio between per-site recombination rate and per-site mutation rate

RM: minimum number of recombination events in the history of the sample

**Fig. 5:**
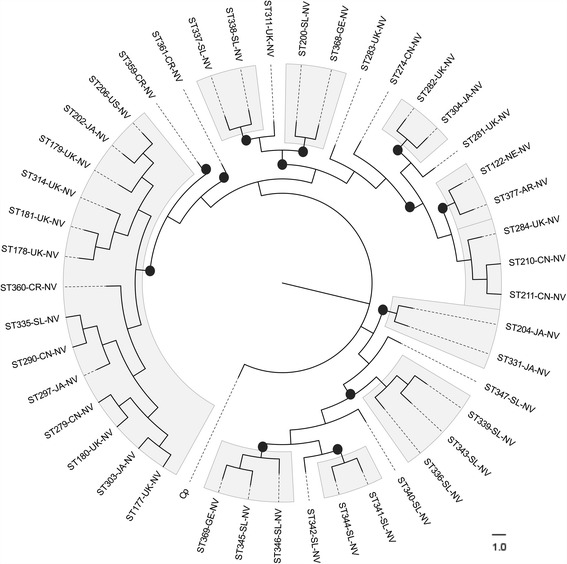
Phylogenetic reconstruction from concatenated sequences of STs grouped as NV. Well-supported clusters are highlighted in grey (n: 10 cluster) and black dots represent bootstrap values ≥ 80.0%

The group of STs having the greatest amount of incongruences regarding topology were those grouped as NV, where well-supported clusters were found for all housekeeping genes. Comparing these findings revealed common grouping patterns for certain groups of STs, possibly indicating new clusters for the members of this group (mainly from SL and JA, such countries having the greatest nucleotide diversity). In order to untangle this hypothesis, we constructed a ML phylogenetic tree with the STs grouped within NV and we found 10 defined clusters and 8 outliers (Fig. 5).

## Discussion

In spite of the serious problem currently represented by CD infection, few studies are available at epidemiological and population structure level and those available are geographically localised [16, 27], meaning that the available data must be analysed to identify infection patterns which might contribute towards developing prevention and control strategies for the disease. Multilocus sequence analysis (MLSA) is now accepted and widely used as a method for defining prokaryotic taxonomy and has a wide range of intra-taxa applications, i.e. estimating genetic diversity [3]. Regarding such usefulness, this analysis was used as an exploratory approach to CD-MLST-db, contributing towards the description of reported ST distribution profiles and geographical source, including the possibility of new clades emergence.

It should be stated that the information available in CD-MLST-db represents a valuable tool for advancing research into CD. However, it presents some disadvantages such as identifying groups of STs which are not available in the exportable files, possibly leading to underestimating the results when analysing this information. Regarding the dataset analysed, three limitations were found regarding the availability of information. The profile/sequence definition database indicated 380 STs reported; however, epidemiological data for 181 STs was not available when verifying them in the isolate database. There was also variation regarding the total amount of data to be analysed for each variable in the breakdown section of the isolate database. Then, in spite of efforts at on-going improvement (evidenced by the updating dates for the databases at increasingly shorter intervals (~10 days)), there is still needed improvement regarding crossed comparison between databases and the datasets available in the exportable files. A third limitation during analysis concerned the non-availability of data regarding traditional typing strategies (PFGE and RAE) nor was data available for genes encoding the two main toxins (*tcdA* and *tcdB*), since such data is fundamental for comparing CD epidemiological profiles [28] and its clinical impact [14], respectively. Data was only found which was related to the presence of genes encoding binary toxin, indicating around 50% of the isolates evaluated being positive for these genes, thereby differing from previously reported data which indicated that only about 23% of CD isolates were positive for this toxin [14].

Most data came from the UK probably due to greater efforts at ascertaining knowledge regarding CD biology and also to rigorous ICD screening strategies [16, 27]. The panorama describing only less than 10% of isolates coming from countries different to the UK (mainly US, CN, JA, SL, AU and IT, together representing 98.1% of isolates) highlights the need for research aimed at describing molecular epidemiology and genetic diversity in other regions. C1 was the most frequent clade, coinciding with that described in the literature reporting relatively high frequency, many STs in this clade being associated with different outbreaks around the world [14]. C1 has been reported in almost all the countries from which the data set herein evaluated have been obtained, except in CR, CL, CO, FR and SP (Fig. 1c). However, these findings could be an indicator of a different population structure in these countries.

Even though most STs analysed were assigned to a clade, the group of 44 isolates which had no clade assigned (NV) came mainly from SL (34.1%) and UK (25.0%), showing the following particularities: they were mostly related to STs which were not in the exportable file, they had the highest diversity indexes (Fig. 2c) and belonged to the group of STs in which most well-supported clusters were found in phylogenetic analysis (Figs. 3, and 5) which might even correspond to additional clades in the traditional CD taxonomy. Additional studies are thus needed for clarifying the existence of such “new clades”, mainly in regions where these STs have been reported grouped as NV, i.e. SL, (Fig. 5); as well as including isolates from other regions around the world, the existence of STs having similar behaviour could also be explored. Incongruences in the classification of some STs (Fig. 3) and clusters within NV group (Fig. 5) confirm the need for genomic epidemiology studies which have been proposed as an alternative for clarifying the phylogenies obtained from different datasets and also the likely emergence of new genetic groups within NV [29].

Analysis of ST distribution revealed the most frequent to be ST1 belonging to the hypervirulent strain (RT027) which belongs to C2; this is interesting as it does not correspond to the most frequently occurring clade. However, C1’s high frequency could be related to a greater amount of STs having been included (Fig. 1b). Temporal distribution analysis showed that whilst most STs have been reported from 2006 to 2009, there has been an exponential increase in the amount of sequences reported for each allele included in the MLST typing scheme for CD during the last few years (Fig. 1d) and that STs having an NV clade report were found to be most different, corresponding to the most recently reported ones, again indicating the need for continuing CD detection and genotyping in different regions around the world. Analysis of diversity by country showed that SL and JA had the most diverse populations, in spite of the amount of isolates reported being low, compared to UK (Fig. 2b). These findings indicated that there could be highly diverse populations; however, the amount of STs analysed must be increased to confirm such hypothesis. Regarding clades traditionally reported for CD, it was found that C2 was the least diverse clade and C5 the most diverse (Fig. 2c). These findings are interesting as C2 includes hypervirulent STs which have been associated with outbreaks of the disease, meaning that study determining the other molecular characteristics of this group of STs must be continued. It was found that the STs called NV for clade classification into CD-MLST-db had considerably higher diversity indexes (Fig. 2c – Additional file 5: Table S3).

Determining intra-taxa diversity identified *adk*, *atpA*, *recA* and *tpi* as conserved genes, thereby fulfilling the requirements for a housekeeping gene used for a MLST scheme*.* However, the other three genes included in the MLST scheme for CD (*sodA*, *dxr* and *glyA*) had a high percentage of sequence variations, corresponding to the majority of informative sites (Additional file 2: Figure 2A and B), as well as having relatively high diversity indexes (Fig. 2a; Additional file 3: Table S1) supported by the reticulated phylogenetic networks (Additional file 8: Figure S5), the strong values of recombination tests observed in Table 1 and high amount of taxon discrimination (Fig. 4; Additional file 9: Figure S6). This means that these housekeeping genes’ usefulness for the MLST scheme must be re-evaluated. Considering that the results were much more marked for *sodA*, excluding this marker from the CD typing scheme should at least be considered.

Allelic plot findings coincided with those reported in the literature where most STs belonging to the 5 clades traditionally accepted for CD belonged to a single cluster for all genes, except C5 in *adk* and *sodA*. This classification was also corroborated when we ran an NJ tree of the total of STs (Fig. 3) despite of the incongruences. It has been described that CD is an organism having clonal population structure and mutational evolution [5]. However, several interesting findings are worth mentioning: i) Incongruences in the clustering of STs across the different clades/clusters when running NJ phylogenetic reconstruction (Fig. 3), ii) Incongruences in the classification of certain STs with some housekeeping genes (mainly *sodA*) indicating the presence of alleles from other clusters in the allelic plot (Fig. 4); iii) The Neighbour-Net phylogenetic networks displayed reticulated topology and foreseen incongruences in the clustering; iv) The quantification of recombination events was positive for the different methods tested by Dnasp 4.0 (Table 1) and v) The further analyses of Clonal Complexes by eBURST showed a tailored sharing of alleles within groups. These strong signals of recombination agreed with previous reports proposing CD as a genetically heterogeneous species [30] which could be associated with genome plasticity (4.1 to 4.3 Mb size and high mobile genetic content elements (11%), mainly conjugative transposons (CTns)) [13]. In addition to these characteristics, homologous recombination events have been identified in CD (acquired intergene spacer mutations and RNA operons used for ribotyping) [31]. The exchange of genetic material could have biological implications since it could be participating in the mobilisation of loci encoding toxins or conferring resistance against antibiotics [14, 32] and also be influencing species diversification which might be the reason for the emergence of hypervirulent strains, associated with the increased impact of CD infection-associated pathologies during the last 15 years [14]. These signals have been already described in other pathogenic bacteria such as *Streptococcus pneumoniae* [33], but the forthcoming studies of the population genomics of CD are required to fulfil this hypothesis.

## Conclusion

This CD-MLST-db mining approach has thus highlighted this database’s great usefulness for describing CD’s STs and clades regarding the diversity of all STs reported to date. The available data, together with an MLSA, has led to advances in describing this pathogen’s population structure from a genetic point of view, confirming that it is mainly a clonal species with stochastic events of recombination, and that groups of different STs could be grouped into additional clades to those which have traditionally been described for CD. Also, it is important to highlight the paradox emergence of new clusters within NV which could represent possible cryptic species and display relevant epidemiological impact in the CD infection.

## Additional files

Additional file 1: Figure S1. Characteristics of housekeeping genes used in MLST scheme. A. Percentages of allele identity per gene. B. The amount of variant sites per housekeeping gene compared to amount of informative sites according to the principle of parsimony. (TIF 1239 kb)
Additional file 2: Figure S2. Polymorphisms throughout the length of the sequences for each housekeeping gen. (XLSX 97 kb)
Additional file 3: Table S1. Diversity indexes by each housekeeping gen. (XLSX 10 kb)
Additional file 4: Table S2. Diversity indexes by country. (XLSX 9 kb)
Additional file 5: Table S3. Diversity indexes by clade. (XLSX 8 kb)
Additional file 6: Figure S3. BURST groups “BG” (n: 14) and STs singleton (n: 24) identified through BURST analysis. (PDF 322 kb)
Additional file 7: Figure S4. Phylogenetic network of concatenated sequences based on Neighbour-Net algorithm. (JPG 402 kb)
Additional file 8: Figure S5. Phylogenetic networks of each gene based on Neighbour-Net algorithm. (PDF 104 kb)
Additional file 9: Figure S6. Phylogenetic reconstructions for each housekeeping gene based on Maximum Likelihood (ML). (PDF 103 kb)

## Abbreviations

*Adk*: *Adenylate kinase*
AR: Argentina
*atpA*: *ATP synthase subunit alpha*
AU: Australia
BG: BURST groups/Clonal Complexes
C: Clade
CA: Canada
CD: Clostridium difficile
CD-MLST-db: CD MLST database
CDT: Binary toxin
CL: Chile
CN: China
CO: Colombia
CR: Costa Rica
CT: Conjugative transposons
DE: Denmark
*Dxr*: *1-deoxy-D-xylulose 5-phosphate reductoisomerase*
FR: France
GE: Germany
*glyA*: *Serine hydroxymethyltransferase*
IT: Italy
JA: Japan
ML: Maximum likelihood
MLSA: Multilocus sequence analysis
MLST: Multilocus sequence typing
NE: Netherlands
NJ: Neighbour-Joining
NV: No value
PFGE: Pulsed-field gel electrophoresis
REA: Restriction endonuclease analysis
*recA*: *Recombinase A*
SL: Slovenia
*sodA*: *Superoxide dismutase*
SP: Spain
ST: Sequence type
TcdA: Toxin A
TcdB: Toxin B
*tpi*: *Triose phosphate isomerase*
UK: United Kingdom
US: United States
