# Supplementary figures and images for: Determining Clostridium difficile intra-taxa diversity by mining multilocus sequence typing databases
Source: BMC Microbiol. 2017 Mar 14;17:62. doi: 10.1186/s12866-017-0969-7 (PMC5348806; doi:10.1186/s12866-017-0969-7)

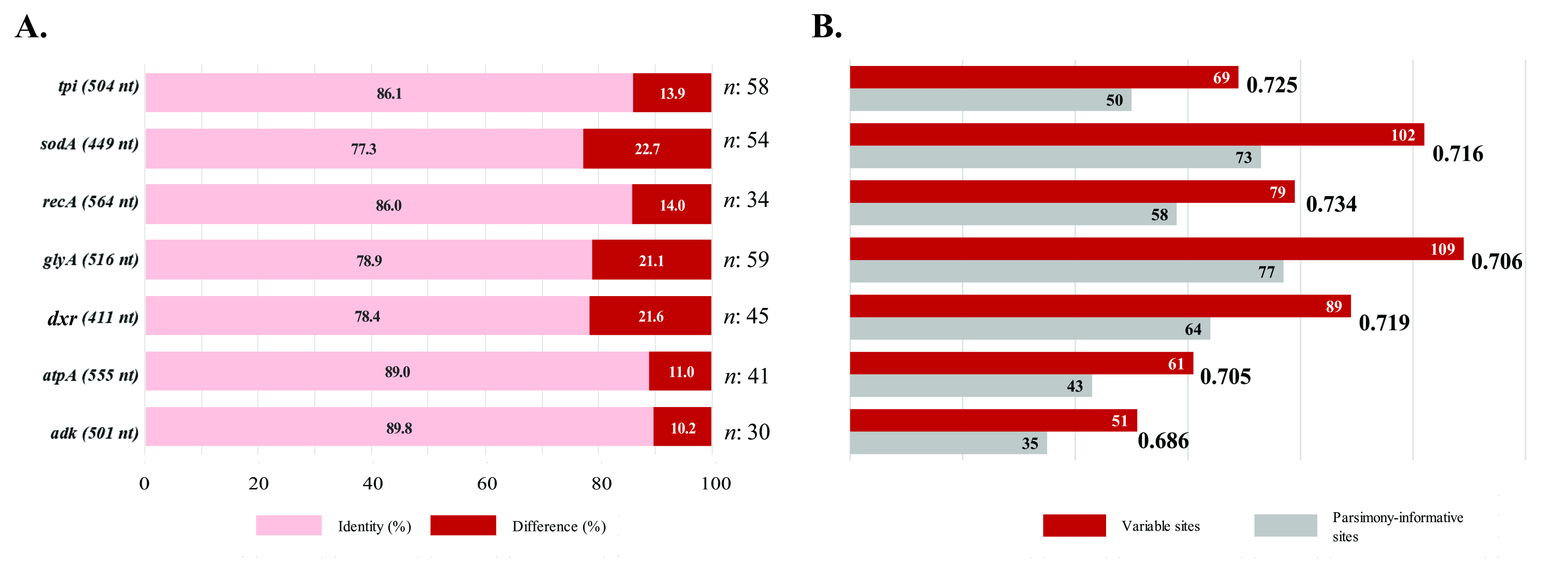

Supplement: Additional file 1: Figure S1. — Characteristics of housekeeping genes used in MLST scheme. A. Percentages of allele identity per gene. B. The amount of variant sites per housekeeping gene compared to amount of informative sites according to the principle of parsimony. (TIF 1239 kb) [file 12866_2017_969_MOESM1_ESM.tif]

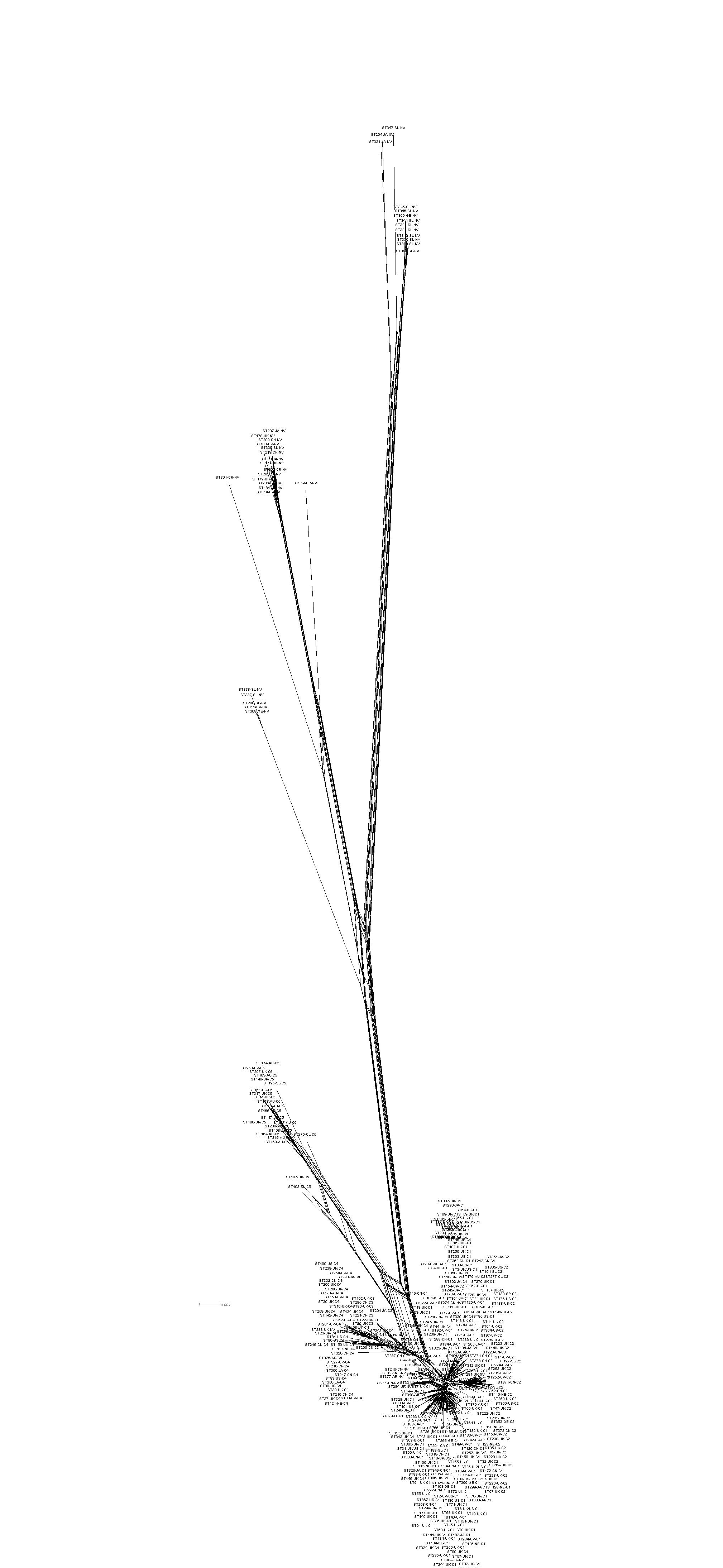

Supplement: Additional file 7: Figure S4. — Phylogenetic network of concatenated sequences based on Neighbour-Net algorithm. (JPG 402 kb) [file 12866_2017_969_MOESM7_ESM.jpg]

*adk*

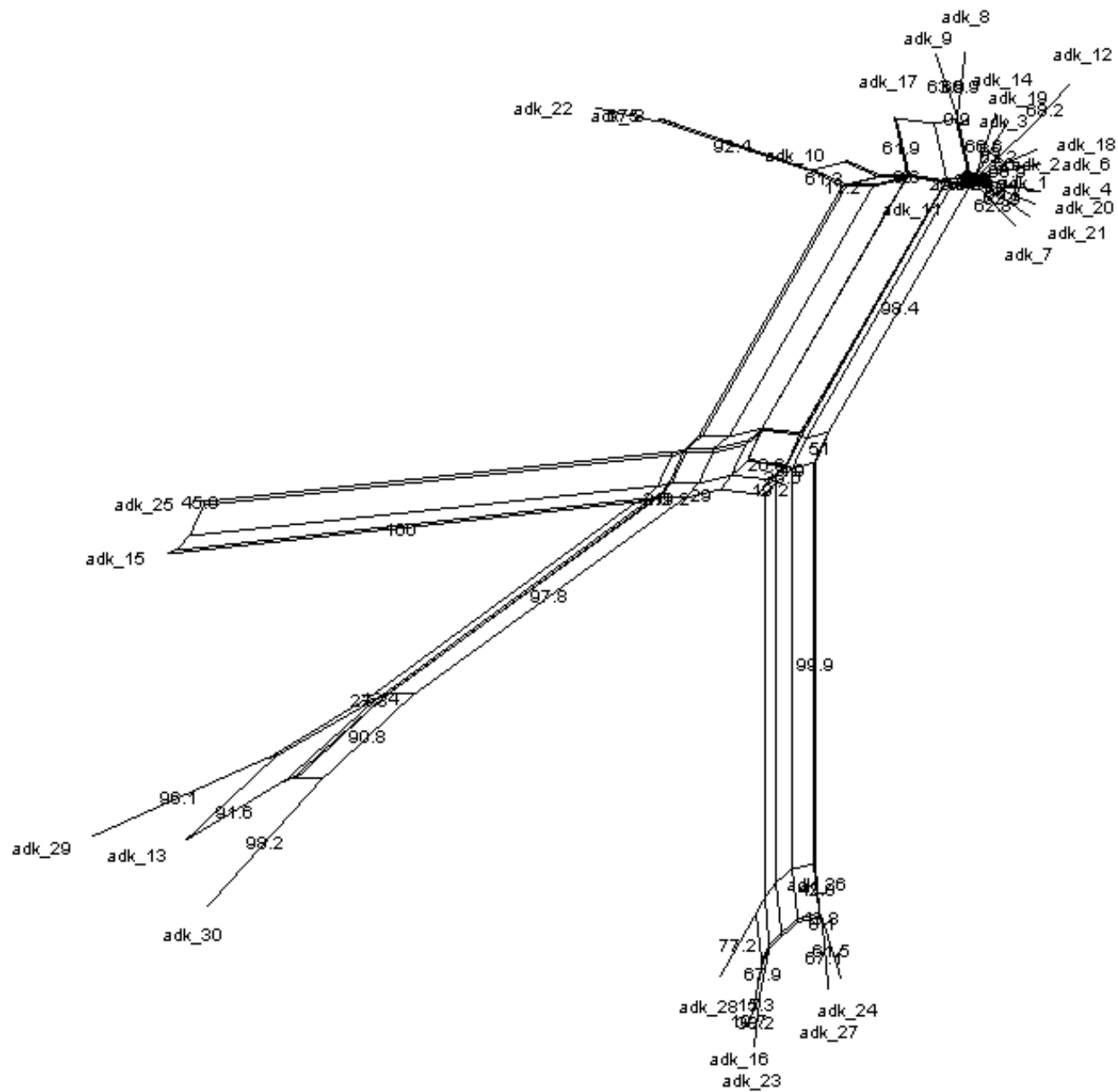





*glyA*

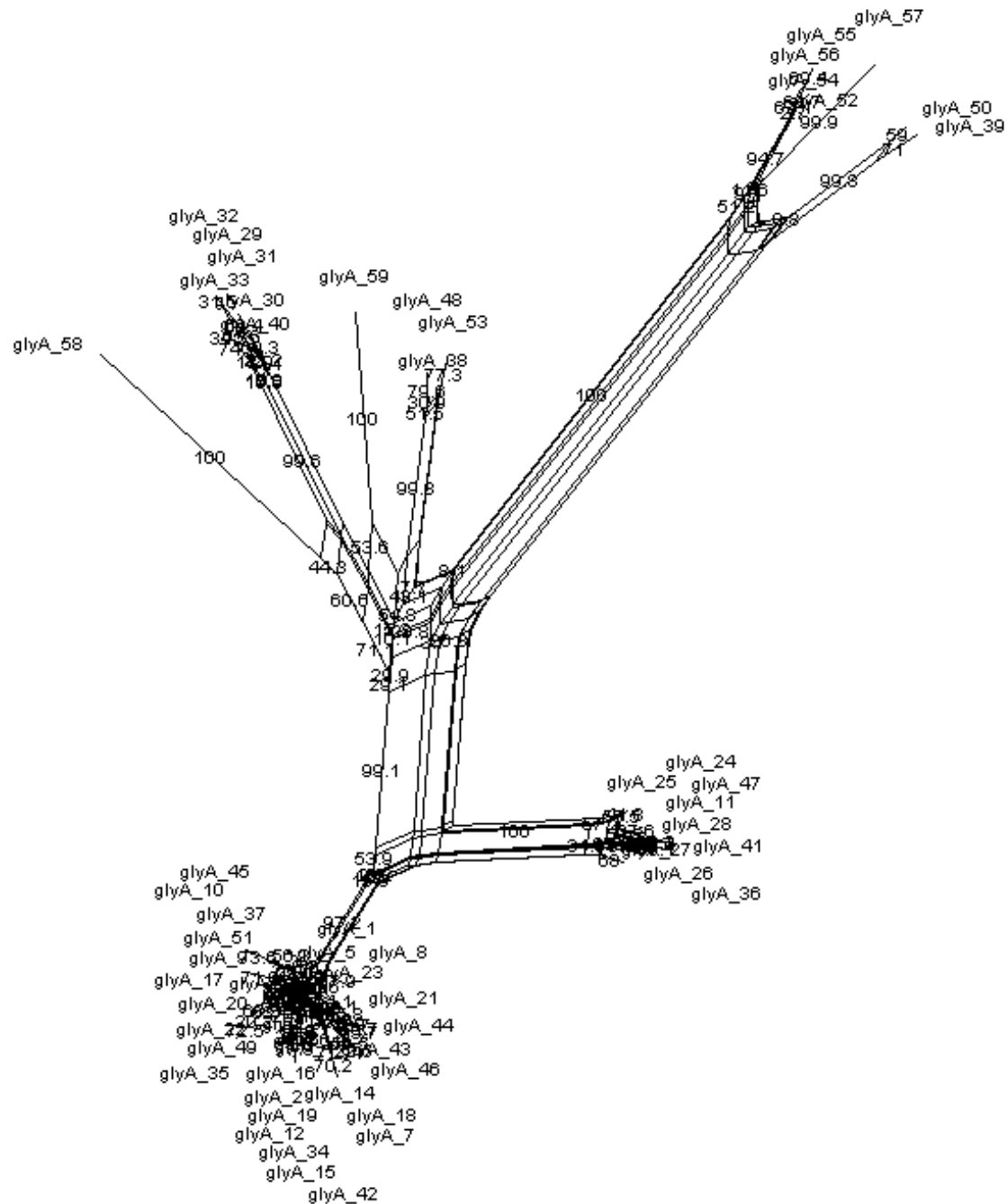

*recA*

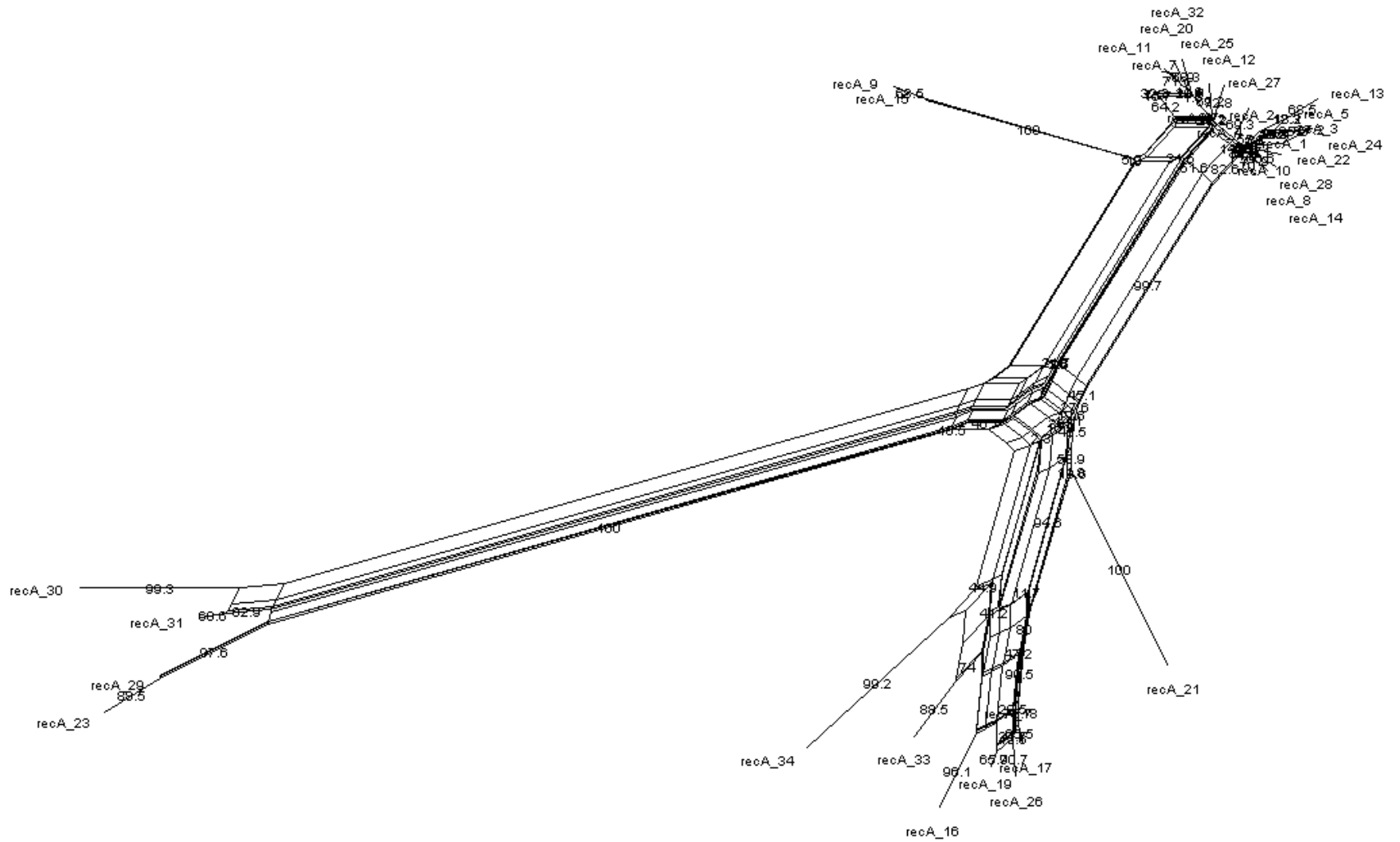

*sodA*

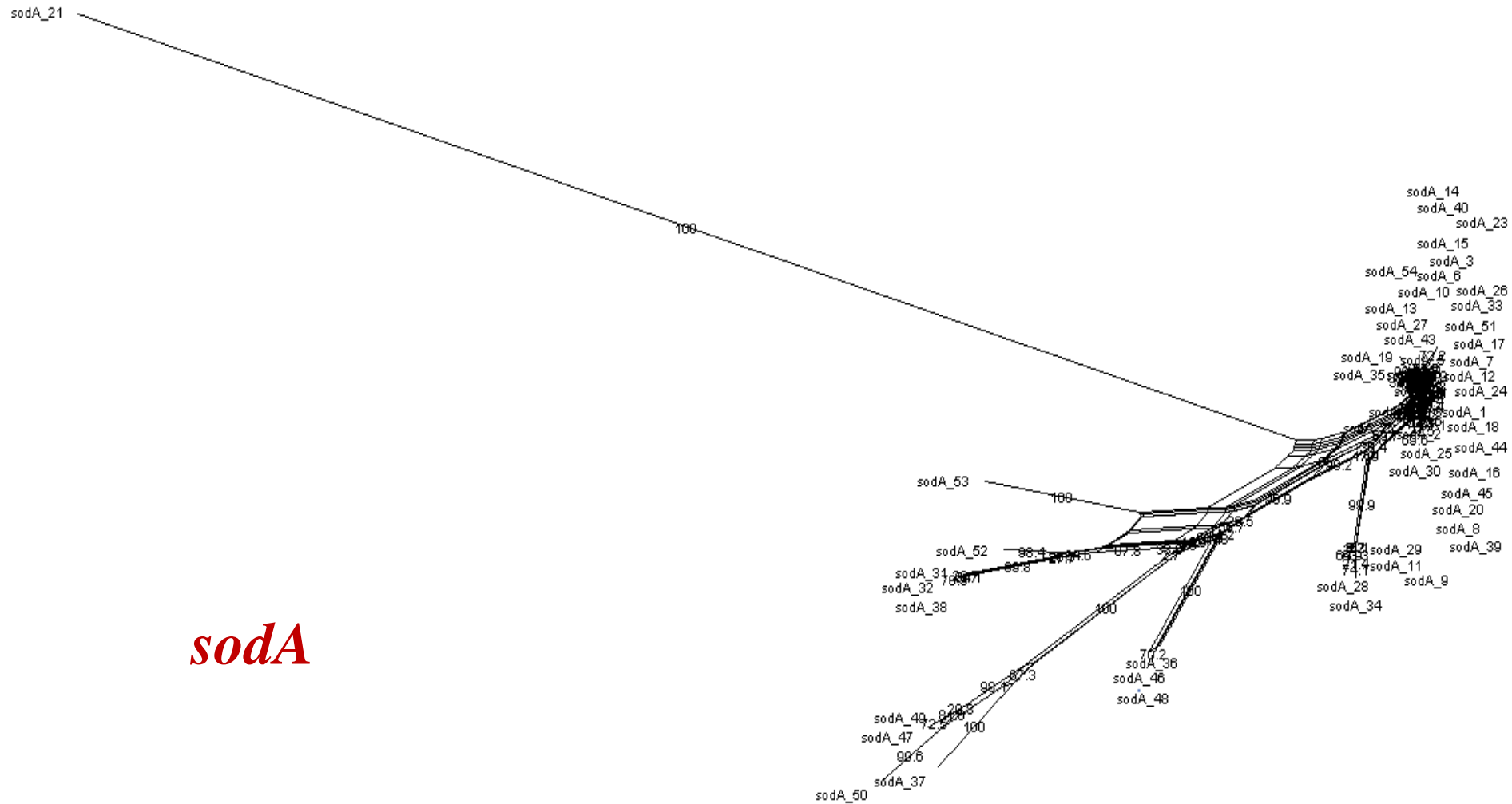

*tpi*

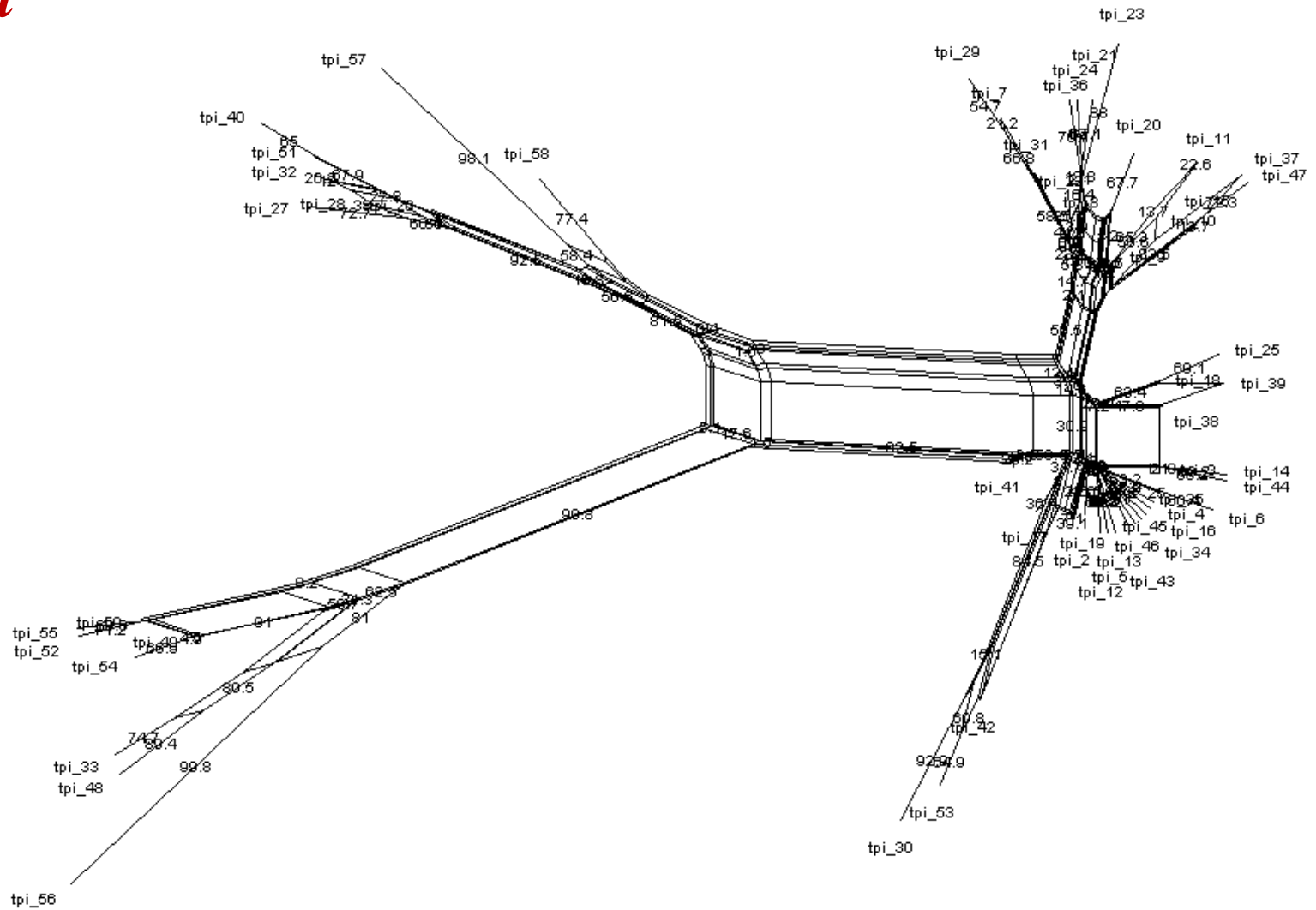

Supplement: Additional file 8: Figure S5. — Phylogenetic networks of each gene based on Neighbour-Net algorithm. (PDF 104 kb) [file 12866_2017_969_MOESM8_ESM.pdf]
