# Supplementary material for: Determining Clostridium difficile intra-taxa diversity by mining multilocus sequence typing databases
Source: BMC Microbiol. 2017 Mar 14;17:62. doi: 10.1186/s12866-017-0969-7 (PMC5348806; doi:10.1186/s12866-017-0969-7)
Supplement: Additional file 6: Figure S3. — BURST groups “BG” (n: 14) and STs singleton (n: 24) identified through BURST analysis. (PDF 322 kb) [file 12866_2017_969_MOESM6_ESM.pdf]

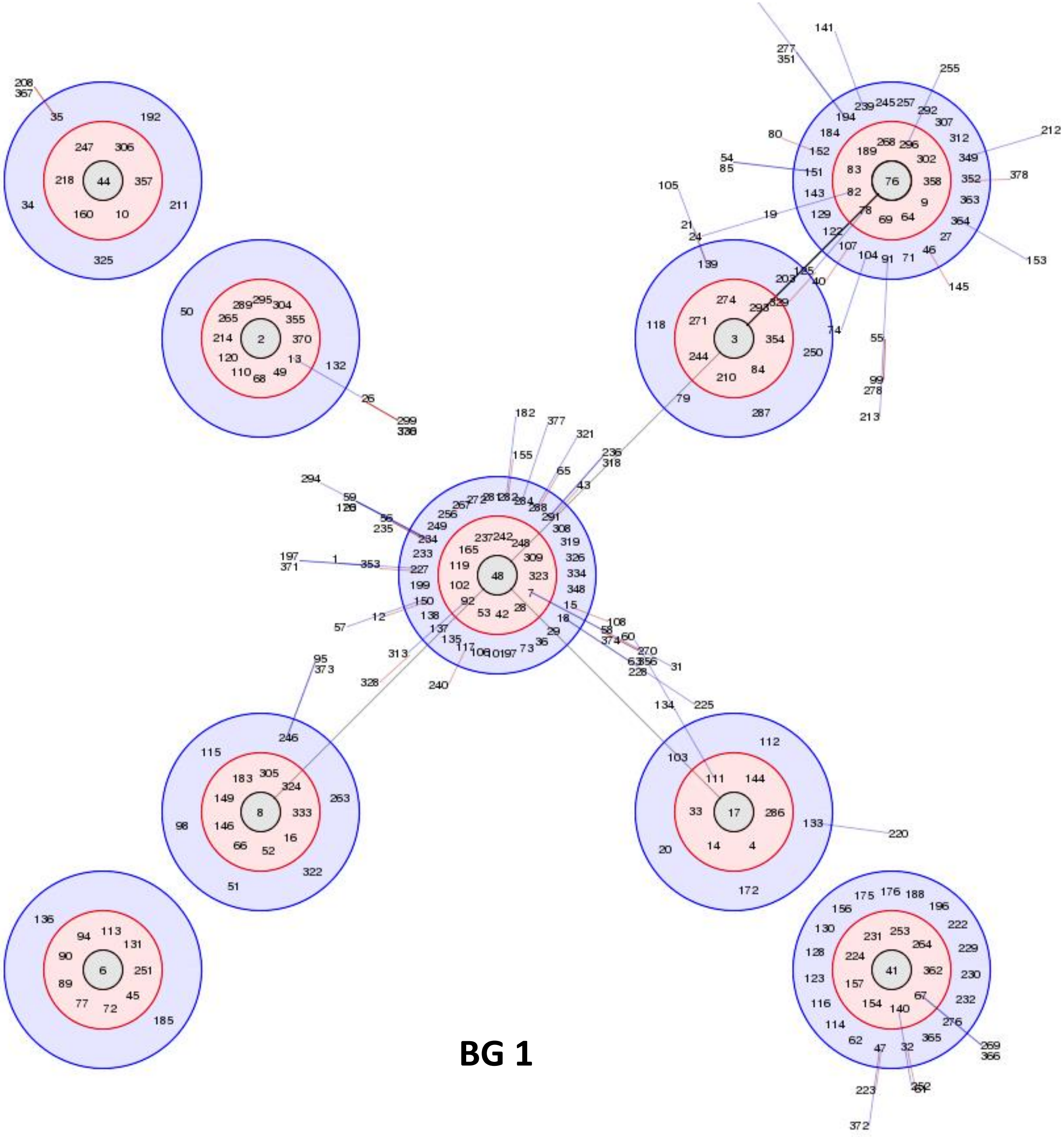

208
367
35
192
44
218
247
306
357
10
160
34
211
325
2
289
295
304
265
355
214
370
120
13
110
68
49
50
132
26
299
336
3
274
271
293
329
354
244
210
84
118
250
79
287
139
203
125
40
76
239
245
257
292
194
184
307
312
349
352
363
364
27
46
71
91
104
107
122
129
143
151
152
189
268
296
302
83
358
82
9
78
69
64
141
277
351
255
212
378
80
54
85
105
21
24
19
153
145
74
55
99
278
213
48
237
242
248
165
309
119
323
102
92
53
42
28
7
267
272
281
282
264
288
291
308
319
326
334
348
15
18
29
36
73
197
106
117
135
137
138
150
199
227
233
284
249
256
182
377
321
155
65
236
318
43
294
59
128
56
235
197
371
1
353
57
12
313
328
240
108
58
374
60
270
63
356
226
31
225
134
95
373
8
183
305
324
149
333
146
16
66
52
115
246
263
98
322
51
17
111
144
33
286
14
4
103
112
133
220
20
172
6
94
113
131
90
251
89
45
77
72
136
185
41
231
253
264
224
362
157
67
154
140
175
176
188
156
196
130
222
128
229
123
230
116
232
114
276
62
365
47
32
269
366
223
252
372

BG 1

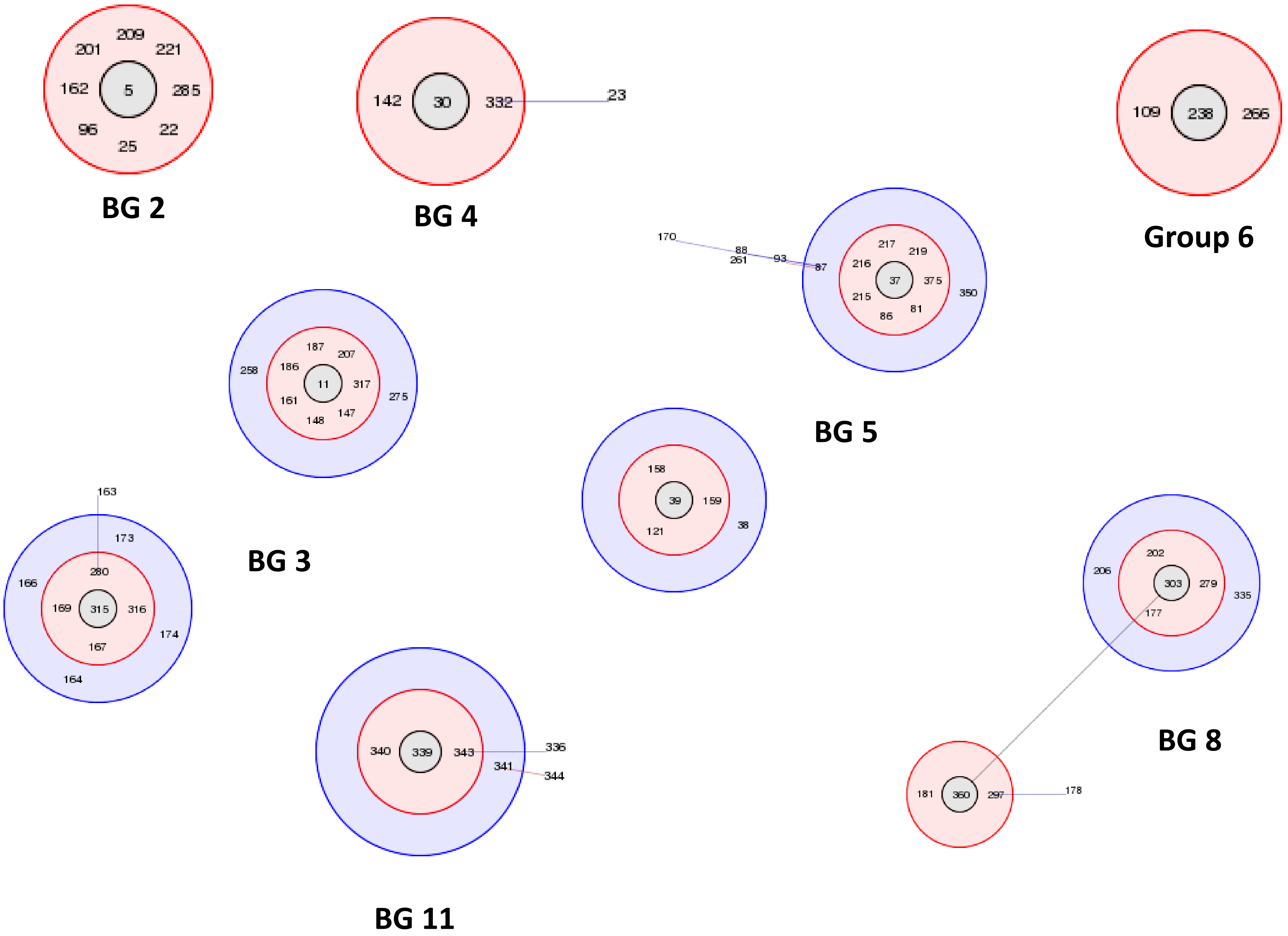

209
201
221
162
5
285
96
22
25
BG 2
142
30
332
23
BG 4
109
238
266
Group 6
170
88
261
93
87
217
219
216
37
375
350
215
86
81
BG 5
187
207
258
186
11
317
161
147
275
148
BG 3
158
39
159
121
38
163
173
280
166
169
315
316
174
167
164
202
206
303
279
335
177
BG 8
340
339
343
336
341
344
BG 11
181
360
297
178

BG 7

ST	Frequency	SLV	DLV	SAT
124	1	3	4	5
127	1	2	4	6
241	1	4	4	4
243	1	4	4	4
254	1	3	7	2
259	1	1	3	8
262	1	1	4	7
273	1	1	2	9
283	1	1	1	10
298	1	3	5	4
310	1	1	3	8
320	1	2	4	6
327	1	0	3	9

BG 9

ST	Frequency	SLV	DLV	SAT
200	1	1	0	
368	1	1	0	

BG 10

ST	Frequency	SLV	DLV	SAT
260	1	0	1	
300	1	0	1	

BG 12

ST	Frequency	SLV	DLV	SAT
337	1	1	0	
338	1	1	0	

BG 13

ST	Frequency	SLV	DLV	SAT
346	1	0	1	
369	1	0	1	

ST	Frequency
75	1
100	1
168	1
171	1
179	1
180	1
190	1
191	1
193	1
195	1
198	1
204	1
205	1
290	1
311	1
314	1
331	1
342	1
345	1
347	1
359	1
361	1
379	1
380	1

24 singlentons
