# Supplementary material for: Determining Clostridium difficile intra-taxa diversity by mining multilocus sequence typing databases
Source: BMC Microbiol. 2017 Mar 14;17:62. doi: 10.1186/s12866-017-0969-7 (PMC5348806; doi:10.1186/s12866-017-0969-7)

adk

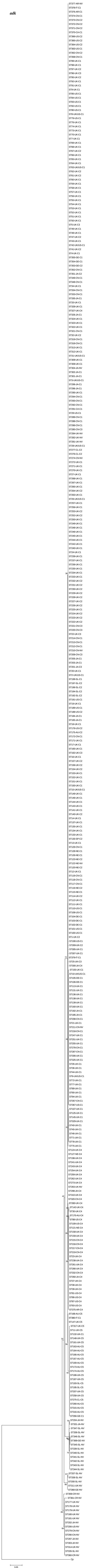

atpA

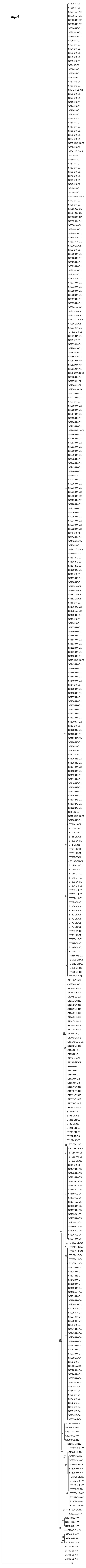

drx

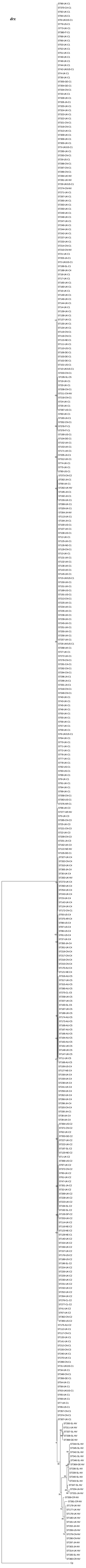

glyA

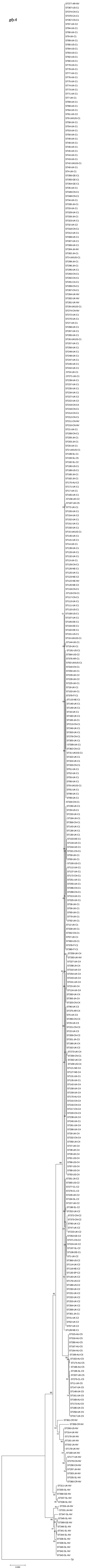

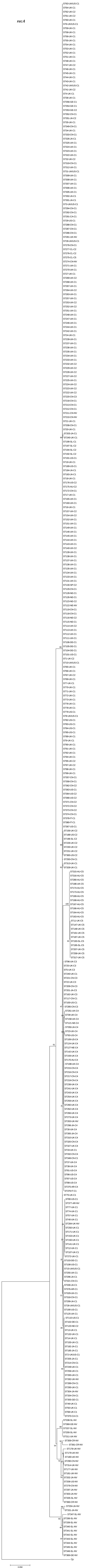
recA

sodA

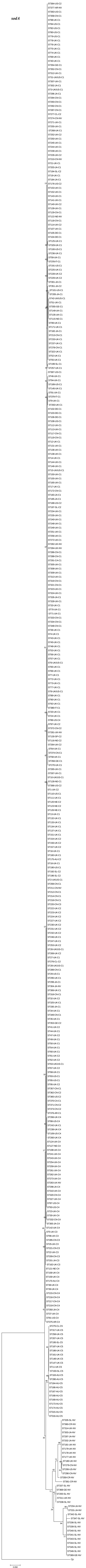

tpi

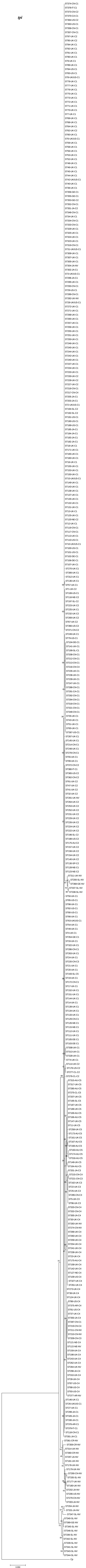

Supplement: Additional file 9: Figure S6. — Phylogenetic reconstructions for each housekeeping gene based on Maximum Likelihood (ML). (PDF 103 kb) [file 12866_2017_969_MOESM9_ESM.pdf]
